# An 8-year cohort study on mosquito population dynamics and meteorological factors in a eastern coastal area of China

**DOI:** 10.1097/MD.0000000000048730

**Published:** 2026-05-15

**Authors:** Xue-yan Zhang, Bo-chao Sun, Xiao-kai Sun, Sheng-lan Zhang, Si-yu Zhu

**Affiliations:** a School of Public Health and Management, Jiangsu Medical College, Yancheng, Jiangsu Province, China; b Department of Chronic Disease Prevention, Yancheng Disease Control and Prevention Center, Yancheng, Jiangsu Province, China; c Department of Chronic Disease Prevention, Yandu Disease Control and Prevention Center, Yancheng, Jiangsu Province, China; d Yancheng Administrative College, Yancheng, Jiangsu Province, China.

**Keywords:** meteorological factors, mosquito surveillance, Yancheng

## Abstract

This study aims to elucidate the dynamic patterns of mosquito species composition and density across seasons and habitats in Yandu District, Yancheng City, from 2016 to 2023 and quantitatively assess the impact of key meteorological factors on population variations, thereby providing a basis for precise risk forecasting of mosquito-borne diseases. Mosquito surveillance was conducted monthly from 2016 to 2023 across 13 sampling sites in urban, rural, and wetland areas of Jiangsu Province using light traps (12 trap-hours per session). Daily meteorological data, including temperature, rainfall, and wind speed, were obtained from local weather stations. Statistical analyses were performed using SAS 9.1 and GraphPad Prism 10. Spearman’s correlation and the Mann-Kendall test were applied to assess relationships and trends. A Generalized Additive Model via Penalized Likelihood (GAMPL) was employed to capture nonlinear effects of meteorological factors on mosquito density, while a Generalized Linear Model (GENMOD) was used to evaluate linear associations. All analyses used a significance level of *α *= 0.05. From 2016 to 2023, a total of 103,901 mosquitoes were captured via light trapping, with an overall density of 15.04 individuals/(lamp·h). These mosquitoes belonged to 8 species, 4 genera, and 2 subfamilies. The dominant species were *Culex pipiens pallens* (65.66%), *Culex tritaeniorhunchus*(18.90%), and *Anopheles sinensis* (10.29%), accounting for 94.85% of the total captures. Monthly mosquito density showed a unimodal distribution, peaking at 29.11 individuals/(lamp·h) in July. Spatially, the annual average mosquito density in wetland parks (45.78 individuals/(lamp·h)) was significantly higher than that in urban environments (*P < .01*). Spearman correlation analysis revealed significant correlations between mosquito density and mean temperature, maximum temperature, minimum temperature, and mean precipitation (PRCP) (all *P < .001*). Generalized linear regression indicated that when temperature exceeded 20°C, each 1°Cincrease was associated with an absolute increase of 1.64 individuals/(lamp·h) in mosquito density (*β = 1.64*). GAMPL analysis demonstrated a significant nonlinear effect of PRCP on mosquito density, with a smoothed component plot showing an inverted “U” shape. Based on these observational findings, mosquito management strategies may benefit from prioritizing wetland and rural zones during July. Furthermore, temperature-precipitation data could potentially inform early warning systems to guide localized control efforts.

## 1. Introduction

In recent years, global climate change and urbanization have significantly changed the ecological habits and geographical distribution of mosquitoes, resulting in a continuous increase in the frequency and transmission risk of mosquito-borne infectious diseases such as dengue fever and Zika virus disease. According to the WHO ‘s 2024 Report on Vector-borne Diseases, vector-borne diseases account for more than 17% of all infectious diseases and cause more than 700,000 deaths per year, of which malaria causes more than 608,000 deaths per year, according to the WHO’s 2024 Report on Vector-borne Diseases.^[1]^Especially in tropical and subtropical regions, seasonal meteorological fluctuations and frequent extreme weather events further aggravate the complexity of mosquito density dynamics.In this context, the analysis of mosquito population dynamics and its correlation mechanism with meteorological factors has become the core issue of accurate early warning and ecological prevention and control of mosquito-borne infectious diseases.

Existing studies generally believe that temperature, humidity and rainfall are the key meteorological factors affecting mosquito densityl.^[2–4]^ Based on the monitoring data of mosquitoes in Yandu District of Yancheng City from 2016 to 2023, combined with meteorological observation data, this study aims to reveal the dynamic response of mosquito species composition and density of different mosquito species (such as Aedes albopictus and Culex pipiens quinquefasciatus) with year, season and habitat in Yandu District of Yancheng City.To explore the contribution of key meteorological factors to the change of mosquito population density. The results of this study can provide a scientific basis for the risk assessment of regional mosquito-borne infectious diseases and lay a theoretical foundation for the formulation of adaptive prevention and control strategies in the context of climate change.

## 2. Materials and methods

### 2.1. Mosquito monitoring data

The monitoring data were from mosquito vector monitoring, and were carried out regularly according to the ‘Work Plan for Mosquito Vector Monitoring in Pilot Areas of Jiangsu Province’ from 2016 to 2023. To ensure standardized sampling and data comparability, Kung Fu Handsome Mosquito Lamps (model LTS-M03) were utilized across all sites, consistently deployed at a constant trap height of 1.5 meters above ground level. In May-October 2016 (July-October 2016), 2017–2020 (June-October 2017), 2021–2023 (May-October 2021–2023), once every half a month, each time for 3 consecutive days, in case of unsuitable weather (rain, wind, etc) delay. In order to guarantee that our research results are representative of the diverse ecological environments where humans may be exposed to mosquitoes, we strategically selected 6 distinct habitat types. In the urban area, 2 residential areas, 2 parks (including street parks) and 2 outdoor places of hospitals were selected. In the rural area, 2 places were selected under the eaves of human houses and near rice fields. In the wetland park, 1 entrance and 1 entrance were selected respectively, and 2 other suitable places in the park (a total of 3 points). The monitoring time began at 19 pm, and the continuous trapping lasted from 12 hours to 7 a.m the next day.

Strict trap calibration and regular maintenance: Pre-monitoring calibration: Before each annual monitoring season, all light traps were calibrated by checking the luminous intensity (using a digital light meter) and power supply stability to ensure uniform trapping efficiency across devices. Routine maintenance: Traps were inspected and cleaned every 2 weeks after monitoring sessions-dust and insect residues on the lamp surface and collection containers were removed, and damaged components (bulbs, batteries) were immediately replaced with identical models. Long-term performance checks: Annually, a third-party technical team verified trap functionality, comparing trapping efficiency with new standard devices to eliminate systematic errors from aging. All participants are trained and qualified to carry out the work. A small number of trap-nights were excluded owing to sporadic equipment malfunction or logistical limitations; however, such incidents were infrequent and distributed proportionally across habitat types. Consequently, we contend that these minor, nonsystematic data gaps do not undermine the robustness of inter-habitat comparisons.

Mosquito density (mosquito/ hour) = number of mosquitoes captured/ (total number of hours captured × number of lights)

### 2.2. Species identification

The identification of insect species was observed under microscope. All evaluators need to be trained and qualified. Morphological identification of insect species referred to “Chinese important medical insect classification and identification.”

### 2.3. Meteorological data

The meteorological data comes from the meteorological department of Yancheng City. The main indicators include: daily average temperature, daily maximum temperature, daily minimum temperature, daily PRCP, daily average pressure and daily average wind speed.

### 2.4. Data analysis

SAS 9.1 (SAS Institute Inc., Cary, NC) statistical software was used for data analysis, and scientific visualization was completed through GraphPad Prism 10 (GraphPad Software, San Diego, CA).In the correlation analysis stage, for the non-normal distribution of mosquito density data and its possible nonlinear relationship with meteorological factors, the Spearman rank correlation coefficient method was used for correlation evaluation.The Mann-Kendall method was used to test the trend of mosquito density, and the Kruskal-Wallis test was used for comparison between groups, with a significant level of *α* = 0.05. Generalized Additive Models were utilized to characterize potential nonlinear relationships between continuous meteorological variables (e.g., temperature, PRCP) and mosquito density. The Generalized Additive Models was fitted using the Newton-Raphson optimization algorithm in SAS (PROC GAMPL), with smoothing parameters for spline terms automatically optimized through generalized cross-validation. Concurrently, Generalized Linear Models with normal distribution and identity link function (PROC GENMOD) were employed to validate the parametric components and assess the linear effects of categorical predictors, including habitat type. All variables were analyzed in their original scales without transformation, using an identity link function under a normal distribution assumption. Model selection was guided by the minimization of standard fit indices, including Akaike’s Information Criterion and Bayesian Information Criterion. The*β*coefficients for parametric terms (e.g., temperature) were interpreted as the absolute change in mosquito density per unit change of the predictor. Collinearity was assessed during preliminary analyses using variance inflation factors, and no significant collinearity was detected among the final predictor set. The model’s adequacy was verified through standard residual diagnostics, including examination of residual vs fitted plots and Q-Q plots, which confirmed no substantial violations of homoscedasticity or normality assumptions.

## 3. Results

### 3.1. Results of mosquito surveillance in 2016–2023

#### 3.1.1. Analysis of population composition

**Figure 1.**
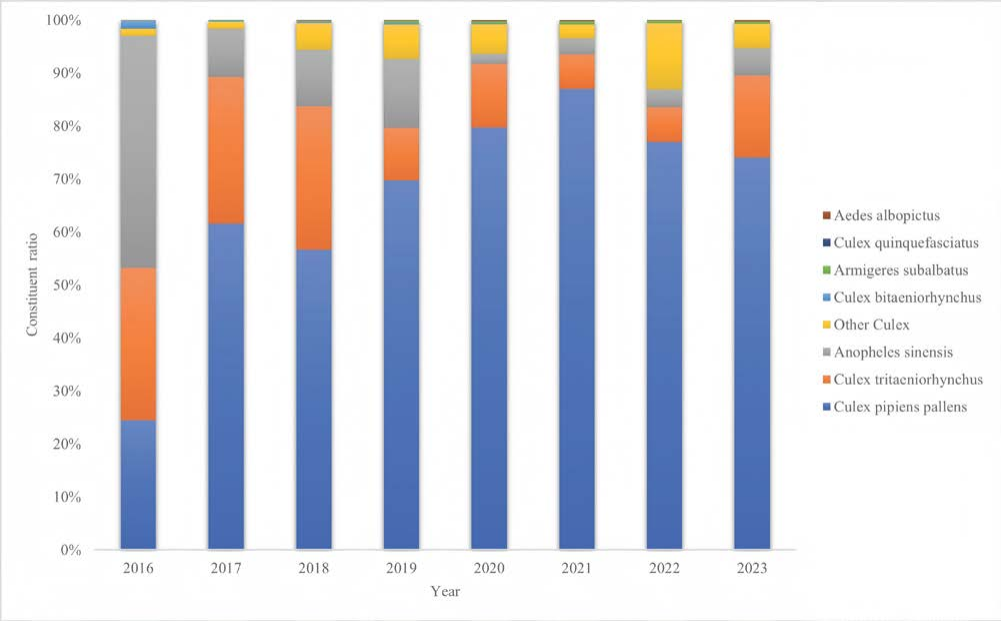
Composition of mosquitoes in Yandu District from 2016 to 2023.

From 2016 to 2023, a total of 103,901 mosquitoes were collected by light trap method in Yandu District of Yancheng City, with a total density of 15.04 mosquitoes/ (lamp·h).The captured mosquitoes belonged to 2 subfamilies, 4 genera and 8 species. Culex pipiens pallens, Culex tritaeniorhynchus and Anopheles sinensis were the dominant mosquito species in the local area, accounting for 65.66%, 18.90% and 10.29% respectively. Except for 2016, the population structure of mosquitoes captured in 2017–2023 was basically stable.The composition ratio of Culex pipiens pallens was maintained between 56.64% and 87.01%, and the proportion was on the rise. The trend test Z = 4.32, *P* < .001.The proportion of Culex tritaeniorhynchus fluctuated between 6.50% and 27.67%, and the proportion gradually decreased, but rebounded to 15.51% in 2023. The proportion of Anopheles sinensis showed a downward trend. Composition of mosquitoes in Yandu District from 2016 to 2023 in Figure 1.

### 3.2. The change trend of mosquito density over time

According to the monitoring data, the mosquito density generally showed a single peak trend in the seasonal months. The mosquito density peaked in July, with a total density of 29.11/(lamp·h), and the lowest in October was 3.45/(lamp·h).In terms of year, the total monitoring density of mosquitoes was the highest in 2017, reaching 23.61/(lamp·h), and the lowest in 2021, reaching 8.02/(lamp·h). In terms of time series, the periodic fluctuation of mosquito monitoring density is obvious.The temporal variation of mosquito density in Yandu District from 2016 to 2023 in Table 1.

**Table 1:** The temporal variation of mosquito density in Yandu District from 2016 to 2023.

Year	May	June	July	August	September	October	Total density
2016	-	-	18.61	27.68	12.30	4.90	16.94
2017	-	8.98	51.03	30.24	16.02	5.60	23.61
2018	-	12.91	40.86	27.14	13.85	1.02	19.33
2019	-	19.49	19.68	14.28	7.65	4.22	13.04
2020	12.82	9.06	15.25	27.24	12.93	1.73	13.15
2021	6.95	9.02	13.68	11.37	5.28	0.97	8.02
2022	5.78	3.49	12.34	16.30	9.71	3.51	8.61
2023	3.05	13.79	52.50	28.75	9.81	5.75	18.55
Total density	7.15	10.86	29.11	22.81	11.10	3.45	15.04

### 3.3. Analysis of mosquito density changing with habitat

The monitoring results from 2016 to 2023 showed that the total monitoring density of wetland parks was the highest, reaching 45.78/(lamp·h), which was significantly higher than that of paddy fields (12.34/(lamp·h)) and residential areas (9.87/(lamp·h)). The total monitoring density of urban hospitals was the lowest, only 1.22/(lamp·h), *H* = 38.21, *P* < .001. The results suggest that the environmental characteristics of different habitats have a significant impact on mosquito density. Rural and wetland environments are more conducive to the reproduction and survival of mosquitoes, while urban environments are relatively unfavorable to the survival of mosquitoes. Mosquito density changed with habitat in Yandu District from 2016 to 2023 in Table 2.

**Table 2:** Mosquito density changed with habitat in Yandu District from 2016 to 2023.

Year	City park	Urban residential area	City hospital	Rural indoor	Outside of rural	Wetland park
2016	1.43	1.65	0.92	27.35	16.71	48.89
2017	4.38	5.06	1.38	12.72	29.76	69.75
2018	3.78	4.34	1.98	11.77	39.32	53.14
2019	6.82	0.90	1.30	16.82	17.12	32.39
2020	4.10	3.97	0.78	6.82	8.65	46.28
2021	3.07	3.04	1.33	6.70	2.95	25.86
2022	1.44	2.07	0.81	3.33	4.49	36.08
2023	3.97	2.02	1.24	15.30	26.78	53.29
Total	3.66	2.93	1.22	11.45	18.25	45.78

### 3.4. Analysis of the influence of meteorological factors on the change of mosquito density

#### 3.4.1. Analysis of relationship

The growth and reproduction of mosquitoes require certain temperature, rain and other conditions. Spearman correlation analysis was used to explore the influence of meteorological factors on mosquito density. The results showed that average temperature, average maximum temperature, average minimum temperature and average PRCP had a certain correlation with mosquito density (*P* < .001), and the correlation between average wind speed and mosquito density was not significant. Spearman correlation analysis of the influence of meteorological factors on mosquito density changes in Table 3.

**Table 3:** Spearman correlation analysis of the influence of meteorological factors on mosquito density changes.

Meteorological factor	*r*	*P*
Mean temperature	0.21	<.001
Mean maximum temperature	0.17	<.001
Mean minimum temperature	0.22	<.001
Average PRCP	0.15	<.001
Average wind speed	0.02	.477

#### 3.4.2. Regression analysis

**Figure 2.**
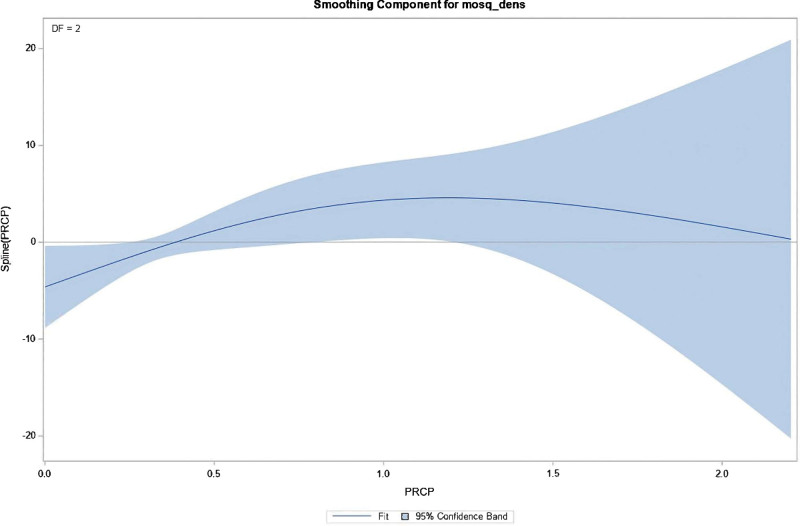
The nonlinear relationship between PRCP and mosquito density.

Considering the multicollinearity among average, maximum, and minimum temperatures, only average temperature, average PRCP, and average wind speed were included in the regression model to analyze their effects on mosquito density. The fitting results of the Generalized Additive Model (GAM) showed that the overall model was statistically significant (*P* < .001). The effective degree of freedom (EDF) for average temperature was 1.00, indicating a significant linear effect on mosquito density. The EDF for average PRCP was 2.01, demonstrating a significant nonlinear effect. As visualized in the smoothed component plot (Fig. 2), this nonlinear association forms an inverted “U” shape. Specifically, within the PRCP range of 0.0 to 1.0, the fitted curve exhibits a positive slope, indicating that moderate precipitation is statistically associated with an increase in mosquito density. Conversely, when PRCP exceeds 1.0, the slope becomes negative, demonstrating a suppressive effect. The narrow 95% confidence band implies high statistical precision for this estimated nonlinear relationship. Average wind speed had no significant effect on the model (*P* = .122). Detailed nonparametric test results of the GAMPL are presented in Table 4.

**Table 4:** Nonparametric test results of GAMPL.

Factor	Effective degree of freedom	*F*	*P*
Mean temperature	1.00	46.31	<.001
Average PRCP	2.01	9.86	<.001
Average wind speed	1.00	2.40	.122

### 3.5. Generalized linear model fitting

The fitting results of the generalized linear model showed that the effect of average temperature on the model was statistically significant, with an estimated value of *β* of 1.64 and a standard error of 0.36. Assuming that other variables remain unchanged, for every 1°C increase in average temperature, the mosquito density increases by an absolute value of 1.64 individuals/(lamp·h) (*β* = 1.64, 95% confidence interval (*95% CI*): 1.52–1.75). The estimated value of *β* of average PRCP is 5.89, *P* = .082, close to 0.05, but it does not reach a significant level, indicating that the impact of PRCP on the model may not be particularly significant, but it cannot be completely ruled out. On the other hand, it also confirms the nonlinear effect of average PRCP on mosquito density in the generalized additive model. The *β* estimate of the mean wind speed is 0.42 (*P* = .866), indicating that the wind speed has no significant effect on the model. GENMOD regression coefficients and test results in Table 5.

**Table 5:** GENMOD regression coefficients and test results.

Parameter	Estimate	Standard error	Wald *95% CI*		Wald chi-square	*P*
Intercept	−28.27	10.83	−49.50	−7.04	6.81	0.009
Mean temperature	1.64	0.36	0.92	2.35	20.19	<.001
Average PRCP	5.89	3.38	−0.74	12.52	3.03	0.082
Average wind speed	0.42	2.49	−4.45	5.30	0.03	0.866

## 4. Discussion

This study showed that the dominant position of Culex pipiens pallens in Yandu District continued to increase, and the proportion of monitored captures increased from 65.66% in 2016 to 87.01% in 2023, which was the main species of mosquitoes in Yandu District. The substantial variations in mosquito density observed across these carefully selected habitats constitute a key finding of the present study. This variability objectively validates the ecological relevance of our site selection strategy and underscores the notable influence of habitat type on mosquito population distribution patterns. The proportion of Anopheles sinensis, which plays an important role in malaria transmission, showed a downward trend. Habitat monitoring showed that wetland parks and rural areas were sites with high mosquito density and should be focused on control. The temporal variation trend of mosquito density showed that July was the peak of mosquito density, which was the same as the results of Zhou et al Therefore, it is recommended to take comprehensive measures to control mosquitoes before the peak of mosquito density in July each year, and to kill overwintering mosquitoes in winter.^[5]^

The correlation analysis of meteorological factors showed that the average temperature, average maximum temperature, average minimum temperature and average PRCP had a certain correlation with mosquito density. Considering the multicollinearity problem, this study selected average temperature, average PRCP and average wind speed into the regression model to analyze the possible impact of meteorological factors on mosquito density. The results showed that the average temperature had a significant linear effect on mosquito density, and the average PRCP had a significant nonlinear effect on mosquito density. The results of generalized linear model fitting show that the influence of average temperature on the model is statistically significant, and the influence of PRCP on the model is not particularly significant. The above results indicate that temperature and PRCP are related to mosquito reproduction, indicating that temperature and PRCP are good variables for predicting mosquito density, which is consistent with the research results of Trawinski and Mackay.^[6]^ It is recommended to further study the establishment of a temperature-precipitation synergistic response model to improve the prediction accuracy.

In summary, this study constructed an 8-year mosquito dynamic database of wetland cities in the Yangtze River Delta, supplemented by generalized additive model and linear regression methods, revealing that temperature had a linear driving effect on mosquito density (EDF = 1.00), while PRCP showed a complex nonlinear response (EDF = 2.01). The research results provide a theoretical basis for mosquito-borne ecological research and mosquito control in coastal wetland cities in the East Asian monsoon region. The generalized additive model revealed a significant nonlinear relationship between PRCP and mosquito density (*P* = .0091), which exhibited an inverted-U pattern. This shape is ecologically interpretable: at low levels of rainfall, availability of standing water is limited, which restricts oviposition and larval development. As PRCP increases, it creates more breeding habitats, leading to a rise in mosquito density. However, beyond a certain threshold, further rainfall may become detrimental, potentially by flushing away larvae and pupae from their breeding sites or diluting the organic nutrients in the water that larvae depend on. Therefore, the observed inverted-U pattern effectively captures the dual role of PRCP, where both insufficient and excessive rainfall can limit mosquito populations, with an optimal range in between. At the same time, this study has several notable limitations. First, the evolution of mosquito insecticide resistance over time may affect the integrity and consistency of the monitoring data. Second, the specific effects of human activities, such as agricultural irrigation, on the local microclimate have not been evaluated, nor have we accounted for potential variations in trap capture rates driven by human behaviors (e.g., localized artificial light pollution or personal use of mosquito repellents). Third, there is an absence of data regarding broader longitudinal environmental and anthropogenic changes. Although our monitoring locations remained strictly stable throughout the 8-year study period (2016–2023), the surrounding micro-environments may have experienced dynamic shifts, including continuous urban growth, modifications in land use, and variations in local pesticide application for vector control. These unmeasured environmental variables act as potential confounding factors that could independently influence mosquito population dynamics. Consequently, while our models robustly capture the associations between meteorological factors and mosquito density, these relationships should be interpreted with the caveat that these unmeasured micro-ecological and anthropogenic changes were not explicitly adjusted for in the current analysis. It is strongly suggested that future follow-up research should pay closer attention to, and comprehensively evaluate, these compounding environmental and behavioral factors alongside meteorological variables.

## Author contributions

**Formal analysis:** Xue-yan Zhang.

**Writing – original draft:** Xue-yan Zhang.

**Writing – review & editing:** Xue-yan Zhang, Si-yu Zhu.

**Conceptualization:** Bo-chao Sun.

**Methodology:** Bo-chao Sun, Xiao-kai Sun.

**Data curation:** Xiao-kai Sun, Sheng-lan Zhang.

**Resources:** Sheng-lan Zhang.
